# EEG Patterns Orienting to Lafora Disease Diagnosis—A Case Report in Two Beagles

**DOI:** 10.3389/fvets.2020.589430

**Published:** 2020-11-05

**Authors:** Helga Demeny, Bogdan Florea, Flaviu Tabaran, Cecilia Gabriella Danciu, Laurent Ognean

**Affiliations:** ^1^Department of Preclinical and Clinical Sciences, Faculty of Veterinary Medicine, University of Agricultural Sciences and Veterinary Medicine, Cluj-Napoca, Romania; ^2^Epilepsy and EEG Monitoring Center, Cluj-Napoca, Romania

**Keywords:** progressive myoclonic epilepsy, EEG, surface EMG, Lafora disease, Beagle

## Abstract

Lafora Disease (LD) is a rare, fatal, late-onset, progressive form of myoclonic epilepsy, occurring in humans and dogs. Clinical manifestations of LD usually include seizures, spontaneous and reflex myoclonus with contractions of the neck and limb muscles. We studied the electroencephalogram (EEG) patterns of two beagles in whom LD was subsequently confirmed by genetic testing. In both cases, the EEG recordings, accompanied by electromyography (EMG), have shown similar uncommon patterns. The hypovoltaged background rhythm was interrupted by waxing “crescendo” polyspikes-slow wave complexes appearing 80–250 ms after the start of intermittent photic stimulation, followed by myoclonic jerks after 80–150 ms. This study highlights the value of EEG in establishing a presumptive diagnosis of LD in dogs.

## Introduction

Lafora disease (LD) is a rare, autosomal, recessive fatal disorder, which affects both humans and dogs. LD has been reported in multiple dog breeds including Basset Hound (1–3), Beagle (3, 4), Miniature Wirehaired Dachshund (5, 6), Miniature and Standard Poodle (1–7), Pointer (8) and Welsh Corgi (3). Dogs affected by LD usually suffer from generalized or focal seizures, myoclonus with contractions of the neck and limb muscles (9). The myoclonus is spontaneous but can also be triggered by sudden noise, bright light or visual stimuli, or sudden movements close to the dog's head. Sleep is also disturbed by hypnic jerking (5, 6, 10–12). Other clinical signs that occur less frequently include ataxia, poor vision/blindness, deafness, dementia, aggression toward people or other dogs, urinary or fecal incontinence, jaw smacking, and flycatching (12). Pathologically, intracellular polyglucosan inclusion bodies (Lafora bodies) are present in the neurons, heart, skeletal muscles, liver, skin, and sweat glands (13).

In dogs, the average age of onset is 7 years (2–5, 11, 12, 14). The first LD symptoms observed are usually represented by spontaneous or provoked myoclonic jerks and generalized tonic-clonic seizures. These myoclonic jerks are often fragmentary, asymmetric, arrhythmic, and increase progressively in frequency, with ataxia and cognitive decline also becoming noticeable in time. As the symptoms worsen, typically the dogs are euthanized a few years after diagnosis (5, 6, 10–12).

The characteristic electroencephalogram (EEG) findings in human patients with early LD include generalized spikes/polyspikes or spike-wave complexes on a slow background activity, during both wakefulness and sleep (15–17). As the disease progresses, the epileptiform activity increases, along with the frequency of the spike-wave complexes and the number of short-duration polyspikes. These findings, in the proper clinical context, are highly suggestive of LD (18). Occipital seizures with visual onset symptoms and myoclonus can easily be triggered using intermittent photic stimulation (IPS) (18).

In dogs, EEG features of LD reported in the literature are rather scarce; bilateral synchronous polyspike-wave paroxysms and erratic myoclonus without EEG correlation have been described in one beagle with LD (19). According to our knowledge, this is the first report of LD in dogs in Romania. We present the cases of two beagles with LD, whose EEG showed spontaneous and induced generalized, periodic epileptic discharges.

## Case Presentations

The two beagles included in the study were not pedigree dogs, were not related, and there was no evidence of consanguinity.

### Case No. 1

An 8-year old, intact male Beagle weighing 20 kg was presented to Demed Small Animal Praxis in Cluj-Napoca, Romania, in November 2018, with a history of a single generalized tonic-clonic seizure 1 month prior to the presentation. The dog was not under antiepileptic treatment at this time. Anamnesis revealed that in the last 2 years, the dog presented myoclonic episodes of the head, neck, and thoracic limb, during wakefulness and certain phases of sleep. These myoclonic episodes appeared spontaneously or were triggered by sudden noises (clap, loud TV volume, car horn), flashes of light, and objects appearing suddenly in the visual field. Consciousness was not impaired.

The dog was normothermic at the moment of presentation. Physical examination revealed no pathological changes besides the myoclonic jerks. These jerks were fragmentary, multifocal or generalized, and were often triggered during physical examination by environmental or internal stimuli. Neurological examination revealed a normal mental status and no cranial nerve deficit. Evaluation of the gait, posture, spinal reflexes, cranial reflexes and muscle tone was unremarkable. The neuroanatomical localization of the seizures was assigned to the forebrain. Based on the clinical history and the physical and neurological findings, the differential diagnosis list included: LD, neuronal ceroid lipofuscinosis, myoclonic epilepsy of unknown origin and structural brain disorder.

As no neurological deficit was observed at the initial neurological examination, structural brain disease was considered less likely, although it could not be entirely excluded. Neuronal ceroid lipofuscinosis was excluded based on the age and breed of the patient, which were not strongly suggestive of this pathology, and the lack of other characteristic clinical signs. Given the chronicity of the myoclonic jerks and the sudden onset epileptic seizures, LD was considered the most likely diagnosis. To confirm or infirm this presumptive diagnosis, genetic testing and muscle biopsy were recommended. The owner agreed to performing a complete blood count (CBC), standard biochemistry investigations, urinalysis, EEG and genetic testing for LD but declined skin/muscle biopsy, brain CT/MRI, and cerebrospinal fluid analysis due to cost considerations. The results of the routine investigations, including hematology, biochemistry and urinalysis, were unremarkable.

EEG was performed with a portable 28-channel equipment (Galileo, EB Neuro S.p.A., Italy). The patient was sedated with medetomidine, 0.05 mg/kg IM (Domitor, Orion Pharma, Finland), and positioned in sternal recumbency in a silent room. An adapted method of EEG electrode placement was used, based on the 10–20 international system for humans (20). Disposable subdermal needle scalp electrodes (13 × 0.4 mm SD52-826-V1, SpesMedica, Italy) were placed to provide a uniform coverage of the scalp; electrode resistance was tested and the electroencephalograph was calibrated prior to and after the EEG recordings. A 10-channel referential montage was set up, using two frontal (F3 left, F4 right), two central (C3 left, C4 right), two temporal (T3 left, T4 right), two parietal (P3 left, P4 right), and two occipital electrodes (O1 left, O2 right), one reference electrode in the vertex (median parietal Pz), and one ground electrode (caudally, on the external occipital protuberance). Filter range settings varied between 0.5 and 50 Hz, sensitivity was set at 10 μV/mm, paper speed at 30 mm/s, with the notch filter on. Common average reference and “double banana” longitudinal bipolar montages were used for reviewing. The setup included one standard electrocardiography (ECG) channel and one surface electromyography (EMG) channel fixed on the right brachiocephalic muscle. EEG recordings lasted for 20 min, and included spontaneous recording and IPS. IPS was performed using a photic stimulator (Flash Led Stimulator SN 00634, EBNeuro, Italy) placed ~30 cm in front of the closed eyes. The planned photic stimulation protocol (21) included periods of varying flash rates stimulation for 10 s, followed by breaks of 5 s. Flash rates were increased in steps of 2 Hz, from 1 to 16 Hz. Due to myoclonus being evoked at certain frequencies, photic stimulation was stopped, as it was not considered ethical to trigger a generalized seizure. Therefore, in the final part of the recording, the stimulation periods were interrupted after 1–3 s, by breaks of few seconds.

**Figure 1:**
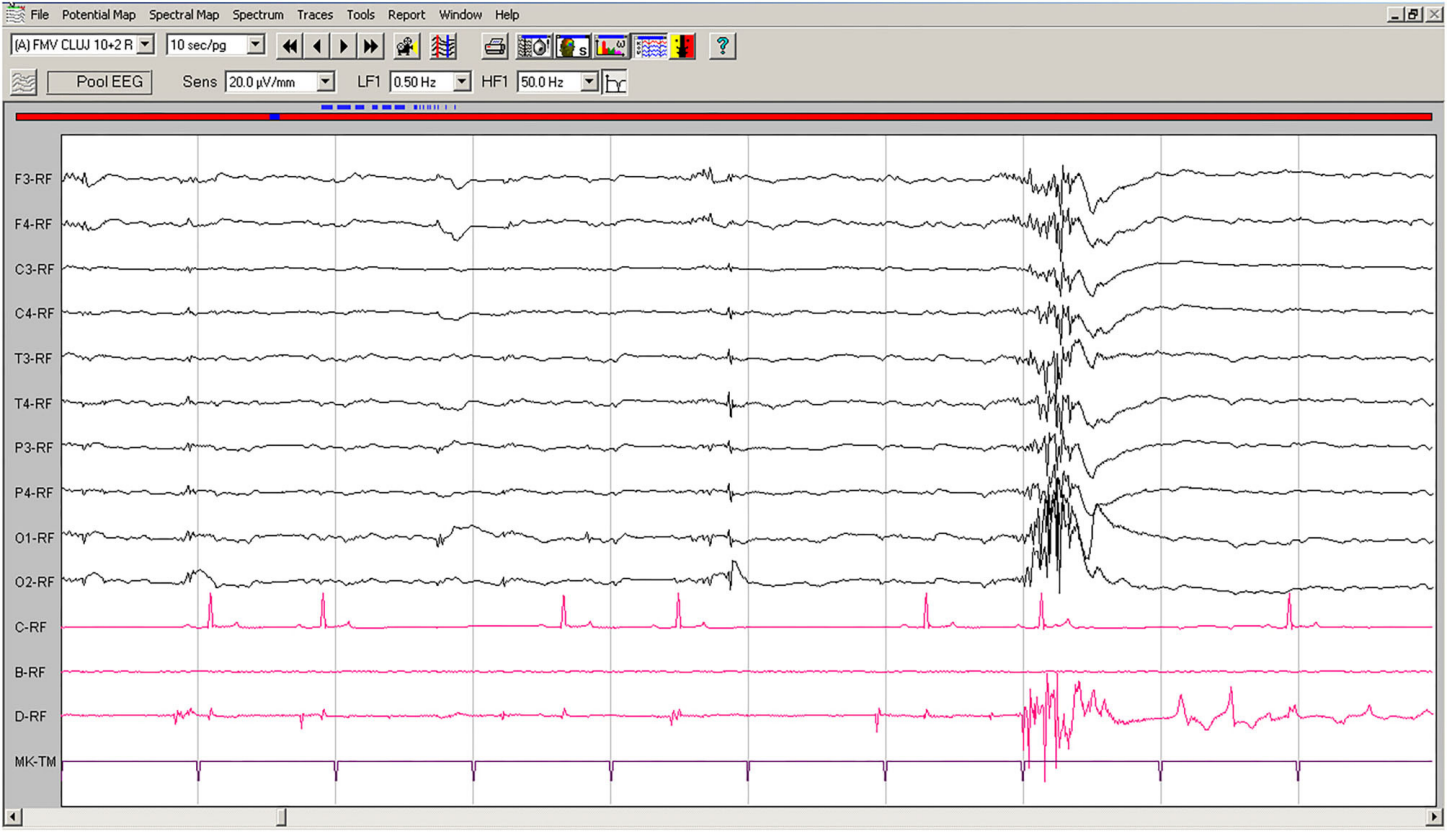
Spontaneous EEG recording of Case No. 1 patient (8-years old male Beagle): Polyspike-slow wave complexes of varying amplitude are seen on all derivations, accompanied by sudden myoclonic contraction of the neck and forelimbs. Low-cut filter: 0.5 Hz; high-cut filter: 50 Hz; speed 10 sec/page; channel C: ECG, bipolar on the chest; channel B: empty; channel D: bipolar, right brachiocephalic muscle. Figure shown at 20 μV/mm. EEG, electroencephalogram; ECG, electrocardiography.

Spontaneous EEG recording revealed solitary spikes (50–250 μV), solitary spike-slow wave complexes, and generalized polyspike-slow wave complexes every 20–40 s (Figure 1). Discharges <100 ms had no clinical correspondent, while discharges >100 ms were accompanied by clonic contractions of the thoracic limb, neck, and generalized myoclonic jerks.

**Figure 2:**
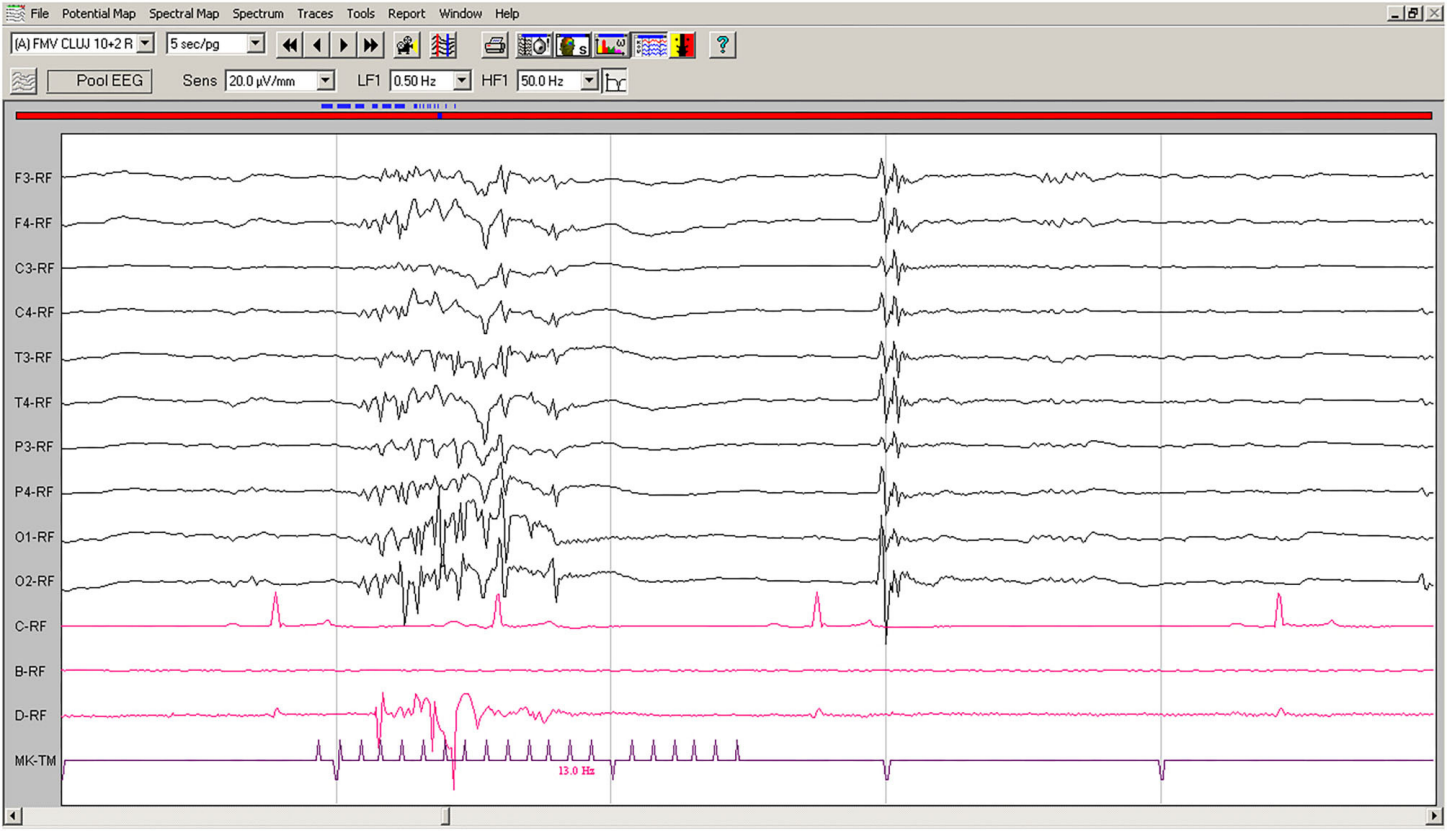
IPS-induced EEG recording of Case No. 1 patient (8-year old male Beagle): IPS at 13 Hz generates polyspikes-slow wave complexes visible on all derivations, accompanied by sudden generalized myoclonic contractions. Low-cut filter: 0.5 Hz; high-cut filter: 50 Hz; speed 5 s/page; channel C: ECG, bipolar on the chest; channel B: empty; channel D: bipolar, right brachiocephalic muscle; MKT channel: photic stimulation marker. Figure shown at 20 μV/mm. IPS, intermittent photic stimulation; EEG, electroencephalogram; ECG, electrocardiography.

On EEG recordings during IPS, the hypovoltaged background rhythm was interrupted by generalized polyspikes followed by a slow wave (Figure 2); IPS was followed after 80–100 ms by cortical polyspikes, correlated with the onset of neck and forelimb contractions.

Genetic testing using LD-PCR was performed at Laboklin Germany. The results confirmed genotype Laf/Laf as homozygous for the mutation causing LD in the NHLCR1-gene (Supplementary Material).

The treatment protocol started following the first consultation, and antiepileptic drugs (AED) added over time for case no. 1 are presented in Table 1.

**Table 1:** Treatment protocol of Case No. 1.

**AED**	**Form of presentation**	**Posology**	**Started**	**Status**
Levetiracetam	500 mg tablets	p.o., 1–1–1	Nov. 2018	Ongoing
Phenobarbital	100 mg tablets	p.o., ½−0–½	Jan. 2019	Ongoing
Clonazepam	2 mg tablets	p.o., ½−0–1	Apr. 2019	Ongoing
KBr	250 mg tablets	p.o.Loading dose: 4–0–3 Maintenance dose: 0–0–2	Oct. 2019	Ongoing

*AED, antiepileptic drug; Kbr, potassium bromide*.

Two months after the initiation of treatment with levetiracetam, the frequency of myoclonic jerks was almost unchanged, and another generalized tonic-clonic seizure occurred. Therefore, phenobarbital was added to the treatment protocol, leading to no generalized seizures being observed over the following period. However, the frequency of myoclonus increased progressively during this time, with episodes occurring both during wakefulness and sleep. Therefore, after 4 months, at the next consultation clonazepam was added to the therapeutic scheme. The following 6 months were seizure-free, with a relative improvement in the frequency of myoclonic jerks, which appeared grouped toward the evening hours. At the subsequent visit, owners reported that the dog had experienced two generalized seizures in 1 week. Phenobarbital and levetiracetam serum concentration were measured and were confirmed to be in the reference therapeutic range (phenobarbital: 20–35 μg/ml, levetiracetam 5–45 μg/ml). As a result, the treatment was supplemented with potassium bromide. Under this treatment protocol, the dog's condition stagnated, generalized seizures became infrequent (1–2 seizures over 3 months), but the frequency of myoclonic contractions increased progressively, until they were noticeable all the time. Video footage provided by the dog's owners depicting myoclonic jerks is available as Supplementary Material.

### Case No. 2

A 7-year old, neutered male Beagle weighing 17 kg was presented to Demed Small Animal Praxis in Cluj-Napoca, Romania, in October 2019, with a history of five generalized seizures over the last year and a half. The owner also described generalized myoclonic episodes that preceded generalized tonic-clonic seizures by hours or even days. According to the owner's description, some myoclonic episodes were triggered by sudden loud noises. The dog was not under antiepileptic treatment at this time.

The dog was normothermic, and the findings of the physical examination were normal at the moment of presentation. Neurological examination indicated normal mental status and no cranial nerve deficit; the gait, posture, muscle tone and spinal reflexes were unaffected. Based on the clinical findings, the neuroanatomical localization of the seizures was assigned to the forebrain. Considering the clinical history and the findings of the physical and neurological examinations, the differential diagnosis list included: LD, neuronal ceroid lipofuscinosis, myoclonic epilepsy of unknown origin and structural brain disorder.

As no neurological deficits were observed at the time of presentation, structural brain disorder was considered as less likely, although it could not be entirely excluded. The age and breed of the patient, along with the lack of other clinical signs, were not strongly suggestive of neuronal ceroid lipofuscinosis. Genetic testing and muscle biopsy were deemed appropriate to confirm or infirm the most likely diagnosis, namely LD. The owner agreed to performing CBC, standard biochemistry investigations, urinalysis, EEG, LD genetic test and skin/muscle biopsy but declined CT/MRI and cerebrospinal fluid analysis due to financial constraints. The results of routine investigations, including hematology, biochemistry, and urinalysis, were normal.

EEG was performed with 10 needle electrodes in the 10–20 system, under sedation, as described for Case No. 1; the only differences were that two channels dedicated to surface EMG were fixed on the right brachioradial and left brachiocephalic muscles, respectively, and that IPS was conducted using flash rates from 1 to 20 Hz, in increments of 2 Hz. Twenty minutes of spontaneous EEG and IPS at different frequencies were recorded. As myoclonus was observed at certain photic stimulation frequencies, the initial IPS protocol was adapted to include stimulation periods of 1–3 s, interrupted by breaks of a few seconds, to avoid triggering a generalized seizure.

**Figure 3:**
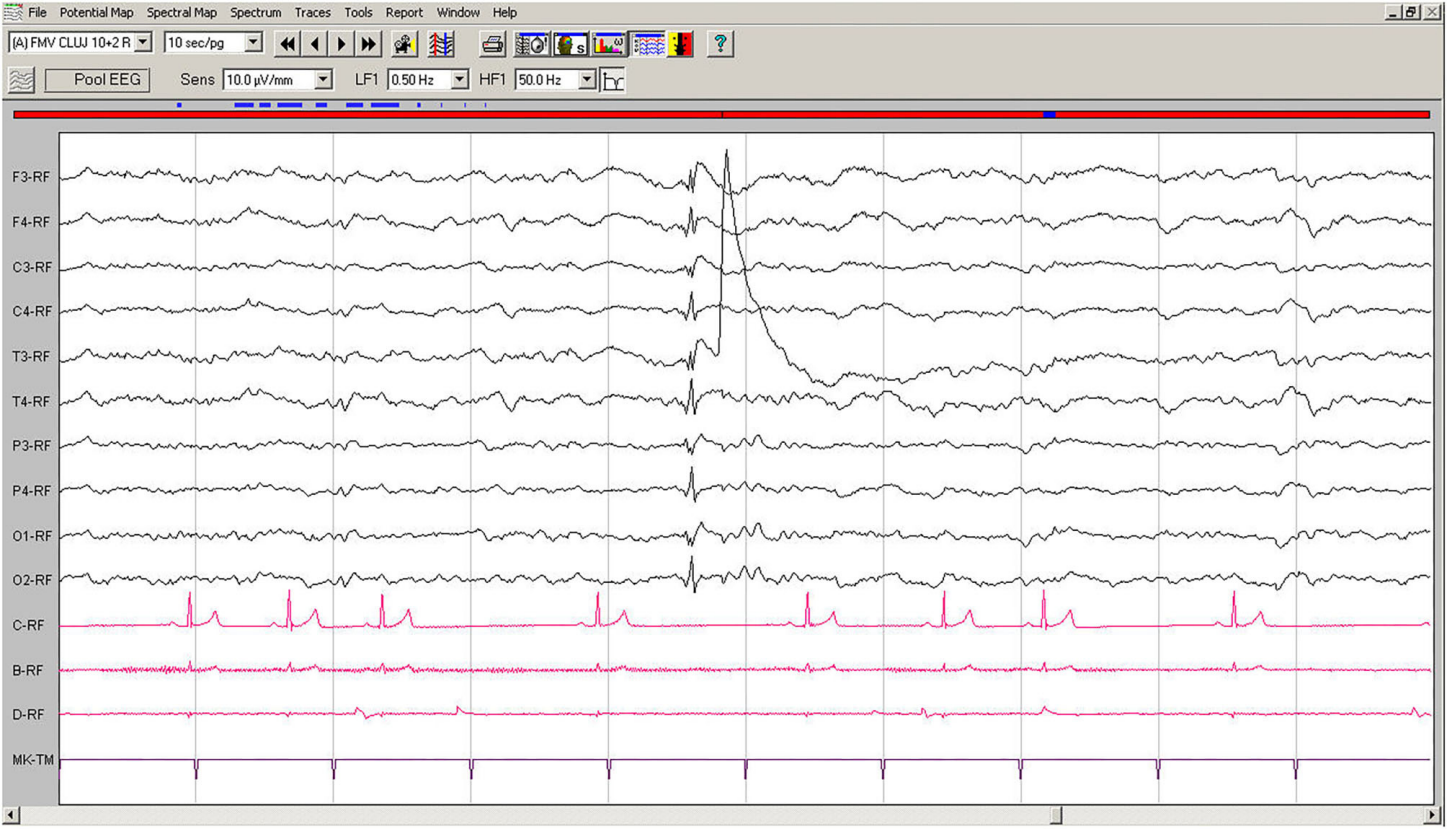
Spontaneous EEG recording of Case No. 2 patient (7-year old male Beagle): In the fifth second, a generalized spike, visible on all derivations, is followed by slow waves. A muscle artifact can be seen after the discharge on T3. Low-cut filter: 0.5 Hz; high-cut filter: 50 Hz; speed 10 sec/page; channel C: ECG: bipolar on the chest, channel B: bipolar, left brachiocephalic muscle; channel D: bipolar, right brachioradialis muscle. EEG, electroencephalogram; ECG, electrocardiogram.

Spontaneous EEG recording revealed an individual spike-slow wave complex which was not accompanied by muscle contraction. Left temporal electrode T3 shows a movement artifact after the discharge (Figure 3).

**Figure 4:**
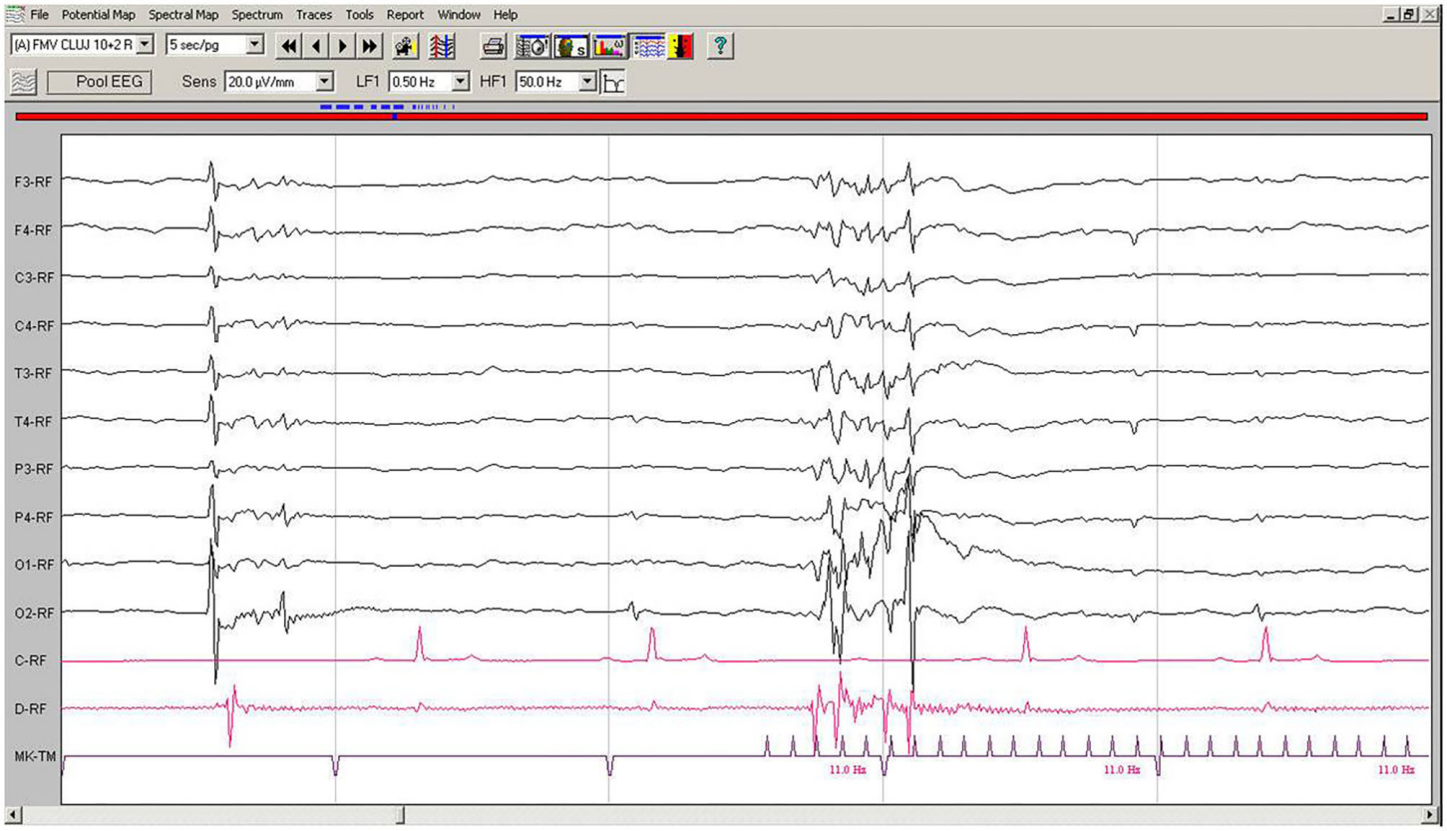
IPS-induced EEG recording of Case No. 2 patient: IPS at 11 Hz generates after 120 ms generalized polyspike-slow wave complexes, followed immediately by the myoclonic contraction on the EMG channel. Low-cut filter: 0.5 Hz; high-cut filter: 50 Hz, speed 5 sec/page; channel C: ECG, bipolar on the chest, channel B: bipolar, left brachiocephalic muscle; channel D: bipolar, right brachioradialis muscle; MKT channel: photic stimulation marker. Figure shown at 20 μV/mm. IPS, intermittent photic stimulation; EEG, electroencephalogram; ECG, electrocardiography; EMG, electromyography.

EEG during IPS showed interruptions of the hypovoltaged background rhythm by generalized polyspikes, followed or not by a slow wave. After 250–600 ms, IPS was followed by cortical polyspikes on EEG, which later on, after 100–150 ms, were correlated with the contraction of the thoracic limb (Figure 4). The amplitude of the background rhythm became lower after the cortical discharge. The left occipital electrode O1 showed a movement artifact after the discharge (Figure 4).

Genetic testing performed at Laboklin Germany confirmed genotype Laf/Laf as homozygous for the mutation causing LD in the NHLCR1-gene (Supplementary Material).

Punch biopsy from the femoral biceps and the overlying skin was obtained. Formalin-fixed sections of the sample were routinely processed into paraffin-wax blocks, sectioned at a 4-μm thickness, and stained with hematoxylin-eosin and periodic acid-Schiff (PAS) stain. Samples were examined under an Olympus BX51 microscope (Olympus, Japan); bright field images were obtained using an Olympus SP 350 digital camera and processed by the Olympus Cell B software.

**Figure 5:**
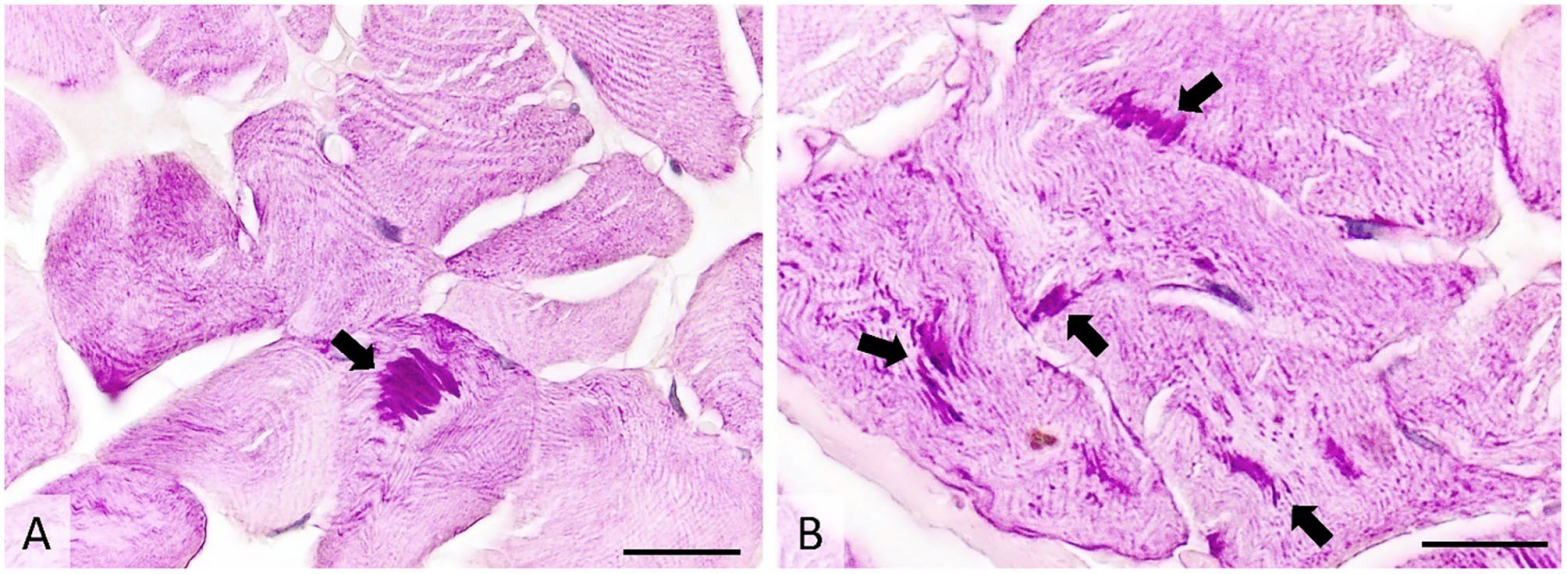
Transverse section of muscle showing many PAS-positive intracytoplasmic inclusions (arrows), occasionally forming subsarcolemmal agglomerations. PAS stain, image **(A)**-ob × 40 (scale bar = 75 μm), image **(B)**-ob × 100 (scale bar = 30 μm). PAS, periodic acid-Schiff.

Histologically, ~10% of the examined skeletal myofibers contained one to several poorly demarcated, intensely PAS-positive subsarcolemmal and intracytoplasmic inclusions measuring 3–50 μm. The inclusions were oval or fibrillar and were more often located in the center of the affected skeletal myofibers than on their periphery. No other significant changes were observed. Inclusions were absent within the sweat glands (Figure 5).

The treatment protocol started after the first consultation, along with the AED added over time for Case No. 2 are presented in Table 2.

**Table 2:** Treatment protocol of Case No. 2.

**AED**	**Form of presentation**	**Posology**	**Started**	**Status**
Phenobarbital	100 mg tablets	p.o., ½−0–½	Oct. 2019	Ongoing
KBr	250 mg tablets	p.o.Loading dose: 3–0–3 Maintenance dose: 0–0–1	Nov. 2019	Ongoing
Levetiracetam	500 mg tablets	p.o., 1–½−1	Feb. 2020	Ongoing
Clonazepam	2 mg tablets	p.o., ½−0–½	Apr. 2020	Ongoing

*AED, antiepileptic drug; Kbr, potassium bromide*.

With a phenobarbital serum concentration level in the therapeutic range, the dog was seizure-free and without any other clinical signs for 2 month. After this time period, despite adequate treatment compliance, the dog had a generalized tonic-clonic seizure, and potassium bromide was added to the treatment protocol. After another 3 months, at the follow-up consultation, the owner complained that myoclonic jerks reappeared in the evenings and while resting, therefore levetiracetam was started. The dog was seizure-free for another 2 months and only occasionally presented reflex myoclonus. Afterwards, the frequency of the myoclonic jerks increased in the second part of the day, leading to the therapeutic scheme being completed by adding clonazepam. Clonazepam lowered the frequency of myoclonus episodes, as in the case of the first patient (Case No. 1). Under this treatment protocol, generalized seizures were absent, and the dog had only a few myoclonic jerks at the end of each day. Video footage provided by the dog's owners depicting myoclonic jerks and a generalized tonic-clonic seizure is available as Supplementary Material.

## Discussion

LD is a progressive neurological condition with non-specific clinical signs that include infrequent generalized tonic-clonic seizures and myoclonus with variable frequency.

Although canine LD cases have been reported in various dog breeds, the relationship between the semiology of the disease and cortical electric activity recorded by EEG has not yet been systematically investigated. In the absence of an epileptic seizure, discrete motor phenomena such as myoclonic twitching or myoclonic contraction of the limbs and neck or even short generalized myoclonia can be the result of infectious diseases such as distemper or pseudorabies, idiopathic meningoencephalomyelitis or drug interactions (22). A differential diagnosis between LD and other neurological or epileptic syndromes can be challenging, especially when the patient presents only generalized seizures and the owner does not notice the reflex myoclonic jerks or when only the myoclonic jerks are observed and the sporadic generalized seizures occur in the absence of the owner.

The relatively late onset of LD in dogs combined with their shorter lifespan means that the disease does not significantly impact life expectancy of canine patients, as it is the case in humans. However, as the symptoms worsen, the owner might opt to have the animal euthanized. Although effective treatment of LD is still lacking, a correct and early diagnosis is the prerequisite of starting an adjusted treatment protocol that has the potential to mitigate the symptoms and enhance the patient's quality of life (23). When appearing during the evolution of a case, generalized tonic-clonic seizures usually confirm cortical involvement and prompt the veterinarian to start antiepileptic medication if the interictal period is <6 months (24). Personalized and frequently adjusted antiepileptic treatment was associated with positive, albeit transient, changes in the two cases described here. After an initial period of improvement in myoclonia, the frequency of the episodes started to increase again in both patients. However, generalized tonic-clonic seizures remained infrequent, and behaviorally the dogs did not show any worsening, suggesting that antiepileptic therapy did improve their quality of life.

EEG can be helpful in orienting the diagnosis and confirming the cortical origin of the myoclonia. As a non-invasive examination method for the diagnosis of functional central nervous system disorders, EEG is considered a routine diagnostic tool for human epilepsy (25). In human LD patients, EEG abnormalities are often detectable before the clinical symptoms appear and are represented initially by an almost normal or slightly slowed background interrupted by generalized or focal paroxysmal activity, usually not influenced by sleep. Occipital discharges arising from a slowed posterior dominant rhythm, corroborated with the clinical signs, can be highly suggestive of LD (23). As the disease progresses, the slowing of background activity becomes evident and frequent bursts of diffuse epileptic discharges appear, along with positive or negative myoclonus and photosensitivity. In the advanced stages, EEG findings include long bursts of diffuse spike-waves and fast polyspikes associated with myoclonic jerks, augmented by low frequency photic stimulation (23).

In contrast to human epilepsy, EEG has not been routinely used in dogs, despite the fact that canine epilepsy is the most common neurological disturbance in veterinary medicine (26). In both cases described here, the dogs with LD presented EEG patterns similar to those described in humans, with solitary spikes or bursts occurring spontaneously or elicited by visual stimuli. The spontaneous, solitary, high amplitude spikes and polyspikes had no clinical correspondent if the discharges were short. However, when they lasted for more than 100 ms, these discharges became robust enough to involve cortical motor areas and generate myoclonus. By performing EEG and EMG simultaneously, we were able to demonstrate that myoclonic jerks in dogs with LD are of cortical origin.

To our knowledge, this is the first report of EEG recordings obtained during IPS in dogs with confirmed LD. During the IPS, the EEG showed a similar pattern of polyspike/slow wave complexes. The first part of the graphic element consists of a polyspike of a frequency not related to the frequency of the photic stimulator, but which appeared regularly with increasing amplitude, and gradually became more and more robust and was followed by muscle contractions at variable time periods. The amplitude of the slow wave that ends the complex was high, but was often hidden by the strong muscle artifact, and the background rhythm became hypovoltaged for at least 3 s. This EEG pattern, with multiple spikes and wave discharges, photosensitivity, multifocal epileptiform discharges, and progressive slowing in background activity is highly suggestive of LD in the human patient (18, 27).

Although confirmation of LD is currently done through genetic testing or muscle and skin biopsy, EEG can also be useful in orienting the diagnosis. EEG findings characteristic of LD have been described in humans (15–18), and we reported here similar changes observed in dogs with subsequently confirmed LD. Results of EEG investigation are obtained more rapidly compared to genetic testing, and the method is less invasive than muscle biopsy. However, besides the equipment, trained personnel are also needed to perform the EEG and correctly interpret the findings. The need for sedation might also act as a deterrent for owners in accepting their companion to undergo EEG.

While our study raises an interesting possibility for using EEG in the diagnosis of LD, it also has several limitations. First, our results are based on only two cases of dogs from a single breed. Second, since the EEG findings reported here were obtained in both dogs only after generalized tonic-clonic seizures have already set in, we cannot state with confidence that EEG modifications precede clinical signs in dogs as they do in humans; therefore, the value of EEG in pre-symptomatic canine patients is still to be evaluated. Third, all reported improvement following antiepileptic treatment was based solely on the accounts of the owners, and therefore the risk of recall bias cannot be completely eliminated. Finally, in both cases EEG was performed under sedation, which could have potentially influenced the recordings; however, sedation of dogs with a low dose of medetomidine has been shown to reduce movement artifacts while allowing clinically valid EEG recordings to be obtained (28).

## Conclusion

Our study illustrates that EEG might be a valuable test for orienting the diagnosis of LD.

Polyspike-slow waves during EEG with IPS with the features described above should invite the clinician to continue further investigations of LD (genetic test and/or muscle biopsy). To the best of our knowledge, this is the first publication that systematically addresses both the spontaneous and IPS-induced EEG features accompanying the subtle motor phenomena in canine LD patients.

## Data Availability Statement

The raw data supporting the conclusions of this article will be made available by the authors, without undue reservation.

## Ethics Statement

Ethical review and approval was not required for the animal study because it was a basic diagnosis procedure for epileptic canine patients. Written informed consent was obtained from the owners for the participation of their animals in this study.

## Author Contributions

HD and BF contributed to the management of cases. HD, BF, and FT contributed to the collection of data. HD, BF, CD, LO, and FT contributed to writing and editing the manuscript and reviewed the final submission. All authors contributed to the article and approved the submitted version.

## Conflict of Interest

The authors declare that the research was conducted in the absence of any commercial or financial relationships that could be construed as a potential conflict of interest.

## Supplementary Material

Supplementary Video 1 Video of Case No. 1 patient depicting myoclonus.

Supplementary Video 2 Video of Case No. 2 patient depicting myoclonus and a generalized tonic-clonic seizure.

## References

[1] Holland JM Davis WC Prieur DJ Collins GH Lafora's disease in the dog. A comparative study. Am J Pathol. (1970) 58:509–30. 5436097 PMC2032841

[2] Kaiser E Krauser K Schwartz-Porsche D Lafora disease (progressive myoclonic epilepsy) in the Bassett hound-possibility of early diagnosis using muscle biopsy? Tierarztl Prax. (1991) 19:290–5. 1653470

[3] Davis KE Finnie JW Hooper PT. Lafora's disease in a dog. Aust Vet J. (1990) 67:192–3. 10.1111/j.1751-0813.1990.tb07754.x 2165776

[4] Hajek I Kettner F Simerdova V Rusbridge C Wang P Minassian B. *NHLRC1* repeat expansion in two beagles with Lafora disease. J Small Anim Pract. (2016) 57:650–2. 10.1111/jsap.12593 27747878 PMC5658008

[5] Fitzmaurice S Rusbridge C Shelton G Minassian B Scherer S Familial myoclonic epilepsy in the miniature wirehaired dachshund. J Vet Intern Med. (2001) 15:72–3.

[6] Lohi H Young EJ Fitzmaurice SN Rusbridge C Chan EM Vervoort M. Expanded repeat in canine epilepsy. Science. (2005) 307:81. 10.1126/science.1102832 15637270

[7] Cusick P Cameron A Parker A Canine neuronal glycoproteinosis-Lafora's disease in the dog. J Am Anim Hosp Assoc. (1976) 12:518–21.

[8] Whitenack DL Neuronal glycoproteinosis (Lafora's disease) in the dog. In: *21st Annual Proceedings*. Madison, WI: American Association of Veterinary Laboratory Diagnosticians (1978). p. 493–6.

[9] Ahonen S Seath I Rusbridge C Holt S Key G Wang T. Nationwide genetic testing towards eliminating Lafora disease from Miniature Wirehaired Dachshunds in the United Kingdom. Canine Genet Epidemiol. (2018) 5:2. 10.1186/s40575-018-0058-8 29610669 PMC5869781

[10] Hegreberg GA Padget GA. Inherited progressive epilepsy of the dog with comparisons of the Lafora's Disease of man. Fed Proc. (1976) 35:1202–5. 1261712

[11] Schoeman T Williams J Wilpe E. Polyglucosan storage disease in a dog resembling Lafora's disease. J Vet Intern Med. (2002) 16:201–7. 10.1111/j.1939-1676.2002.tb02356.x 11899039

[12] Swain L Key G Tauro A Ahonen S Wang P Ackerley C Lafora disease in miniature wirehaired dachshunds. PLoS ONE. (2017) 12:e0182024 10.1371/journal.pone.0182024 28767715 PMC5540395

[13] Jain RS Gupta A Gupta PK Agrawal R Periodic electroencephalogram discharges in a case of Lafora body disease: an unusual finding. Ann Indian Acad Neurol. (2016) 19:269–71. 10.4103/0972-2327.176862 27293346 PMC4888698

[14] Tomchick TL Familial Lafora's disease in beagle dog. Fed Proc Fed Am Soc Exp Biol. (1973) 32:821.

[15] Acharya JN Satishchandra P Asha T. Lafora Disease in South India: a clinical, electrophysiologic, and pathologic study. Epilepsia. (1993) 34:476–87. 10.1111/j.1528-1157.1993.tb02588.x 8389290

[16] Ferlazzo E Canafoglia L Michelucci R Gambardella A Gennaro E PasiniE. Mild Lafora disease: clinical, neurophysiologic, and genetic findings. Epilepsia. (2014) 55:e129–33. 10.1111/epi.12806 25270369

[17] Yen C Beydoun A Drury I. Longitudinal EEG studies in a kindred with Laforadisease. Epilepsia. (1991) 32:895–9. 10.1111/j.1528-1157.1991.tb05548.x 1743163

[18] Araya N Takahashi Y Shimono M Fukuda T Kato M Nakashima M. A recurrent homozygous *NHLRC1* variant in siblings with Lafora disease. Hum Genome Var. (2018) 5:16. 10.1038/s41439-018-0015-9 30083360 PMC6043589

[19] Gredal H Berendt M Leifsson PS. Progressive myoclonus epilepsy in a beagle. J Small Anim Pract. (2003) 44:511–4. 10.1111/j.1748-5827.2003.tb00113.x 14635965

[20] Jasper HH Report of the committee on methods of clinical examination in electroencephalography. Electroencephalogr Clin Neurophysiol. (1958) 10:370–5. 10.1016/0013-4694(58)90053-1

[21] Brauer C Kästner SBR Schenk HC Tünsmeyer J Tipold A. Electroencephalographic recordings in dogs: Prevention of muscle artifacts and evaluation of two activation techniques in healthy individuals. Res Vet Sci. (2011) 90:306–11. 10.1016/j.rvsc.2010.06.004 20591453

[22] Webb AA McMillan C Cullen CL Boston SE Turnbull J Minassian BA. Lafora disease as a cause of visually exacerbated myoclonic attacks in a dog. Can Vet J. (2009) 50:963–7. 19949558 PMC2726025

[23] Turnbull J Tiberia E Striano P Genton P Carpenter S Ackerley CA. Lafora disease. Epileptic Disord. (2016) 18:38–62. 10.1684/epd.2016.0842 27702709 PMC5777303

[24] Bhatti SF De Risio L Muñana K Penderis J Stein V Tipold A. International Veterinary Epilepsy Task Force consensus proposal: medical treatment of canine epilepsy in Europe. BMC Vet Res. (2015) 11:176. 10.1186/s12917-015-0464-z 26316233 PMC4552371

[25] Flink R Pedersen B Guekht AB Malmgren K Michelucci R Neville B. Guidelines for the use of methodology in the diagnosis of epilepsy. Acta Neurol Scand. (2002) 106:1–7. 10.1034/j.1600-0404.2002.01361.x 12067321

[26] Ekenstedt KJ Oberbauer AM. Inherited epilepsy in dogs. Top Companion Anim Med. (2013) 28:51–8. 10.1053/j.tcam.2013.07.001 24070682

[27] Smith SJ. EEG in the diagnosis, classification, and management of patients with epilepsy. J Neurol Neurosurg Psychiatry. (2005) 76:ii2–ii7. 10.1136/jnnp.2005.069245 15961864 PMC1765691

[28] Tepper LC Shores A Electroencephalographic recordings in the canine: effects of low dose medetomidine or dexmedetomidine followed by atipamezole. Open J Vet Med. (2014) 4:7–13. 10.4236/ojvm.2014.42002

