# Supplementary material for: EEG Patterns Orienting to Lafora Disease Diagnosis—A Case Report in Two Beagles
Source: Front Vet Sci. 2020 Nov 5;7:589430. doi: 10.3389/fvets.2020.589430 (PMC7674959; doi:10.3389/fvets.2020.589430)
Supplement: Supplementary file 3 [file Data_Sheet_1.PDF]

CARE Checklist of information to include when writing a case report

CARE case report guidelines

(cc) BY-NC-ND

Topic	Item	Checklist item description	Reported on Line
Title	1	The diagnosis or intervention of primary focus followed by the words “case report”	1-2
Key Words	2	2 to 5 key words that identify diagnoses or interventions in this case report, including "case report"	12
Abstract (no references)	3a	Introduction: What is unique about this case and what does it add to the scientific literature?	15-23
	3b	Main symptoms and/or important clinical findings	15-23
	3c	The main diagnoses, therapeutic interventions, and outcomes	15-23
	3d	Conclusion—What is the main “take-away” lesson(s) from this case?	22-23
Introduction	4	One or two paragraphs summarizing why this case is unique (**may include references**)	26-55
Patient Information	5a	De-identified patient specific information.	62-63, 142-143
	5b	Primary concerns and symptoms of the patient.	63-69, 144-147
	5c	Medical, family, and psycho-social history including relevant genetic information	58-59, 63-69, 144-147
	5d	Relevant past interventions with outcomes	NA
Clinical Findings	6	Describe significant physical examination (PE) and important clinical findings.	70-78, 148-150
Timeline	7	Historical and current information from this episode of care organized as a timeline	124-138, 195-205
Diagnostic Assessment	8a	Diagnostic testing (such as PE, laboratory testing, imaging, surveys).	90-121, 163-192
	8b	Diagnostic challenges (such as access to testing, financial, or cultural)	85-89, 159-161
	8c	Diagnosis (including other diagnoses considered)	75-84, 151-158
	8d	Prognosis (such as staging in oncology) where applicable	NA
Therapeutic Intervention	9a	Types of therapeutic intervention (such as pharmacologic, surgical, preventive, self-care)	124-138, 195-205
	9b	Administration of therapeutic intervention (such as dosage, strength, duration)	Tables 1 and 2
	9c	Changes in therapeutic intervention (with rationale)	124-138, 195-205
Follow-up and Outcomes	10a	Clinician and patient-assessed outcomes (if available)	124-138, 195-205
	10b	Important follow-up diagnostic and other test results	NA
	10c	Intervention adherence and tolerability (How was this assessed?)	132-133, 192
	10d	Adverse and unanticipated events	NA
Discussion	11a	A scientific discussion of the strengths AND limitations associated with this case report	255-283
	11b	Discussion of the relevant medical literature **with references**.	209-254
	11c	The scientific rationale for any conclusions (including assessment of possible causes)	247-254
	11d	The primary “take-away” lessons of this case report (without references) in a one paragraph conclusion	286-291
Patient Perspective	12	The patient should share their perspective in one to two paragraphs on the treatment(s) they received	229-233
Informed Consent	13	Did the patient give informed consent? Please provide if requested	Yes ☒ No ☐
