# Supplementary figures and images for: EEG Patterns Orienting to Lafora Disease Diagnosis—A Case Report in Two Beagles
Source: Front Vet Sci. 2020 Nov 5;7:589430. doi: 10.3389/fvets.2020.589430 (PMC7674959; doi:10.3389/fvets.2020.589430)

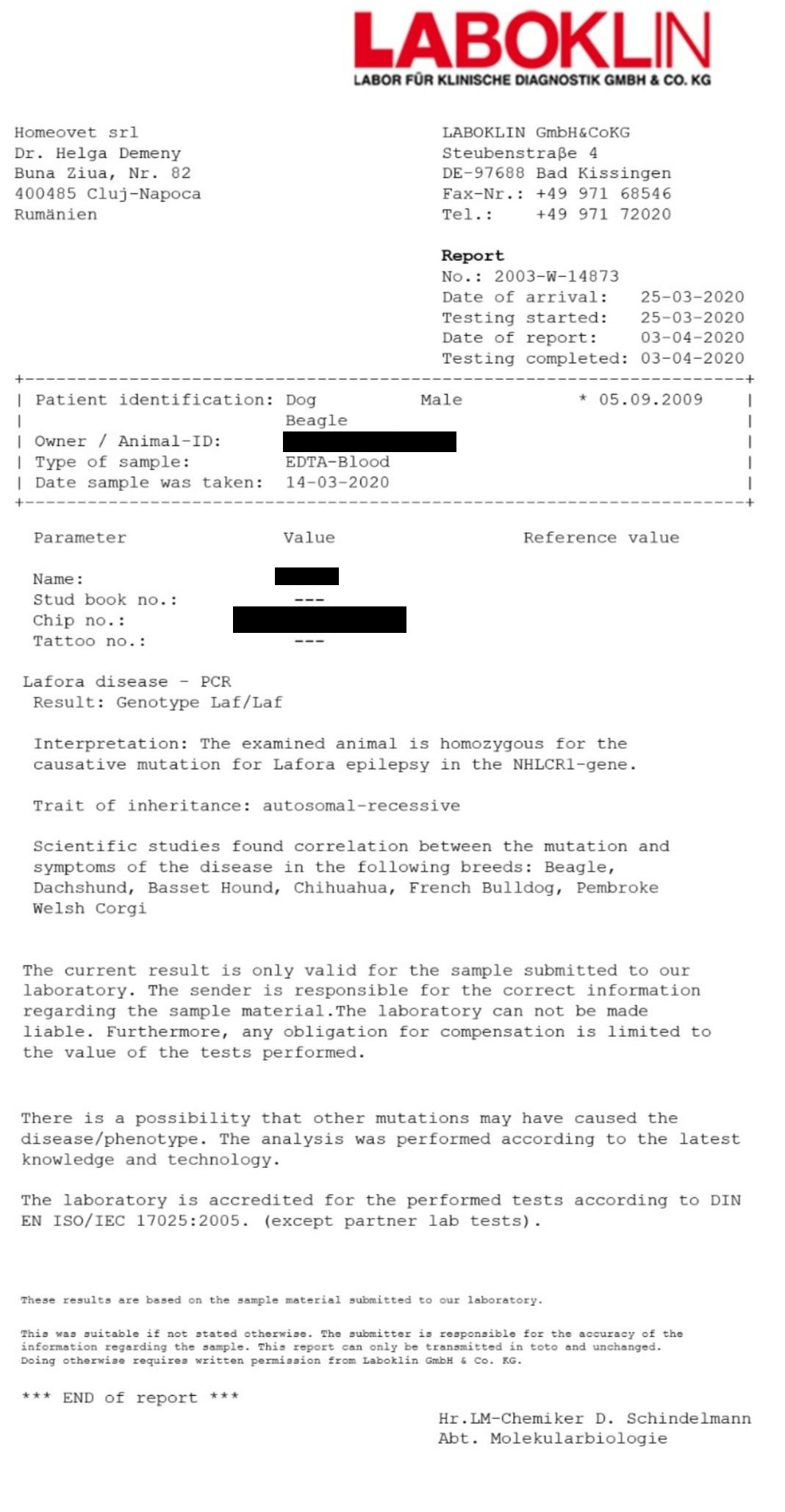

Supplement: Supplementary file 4 [file Image_1.JPEG]

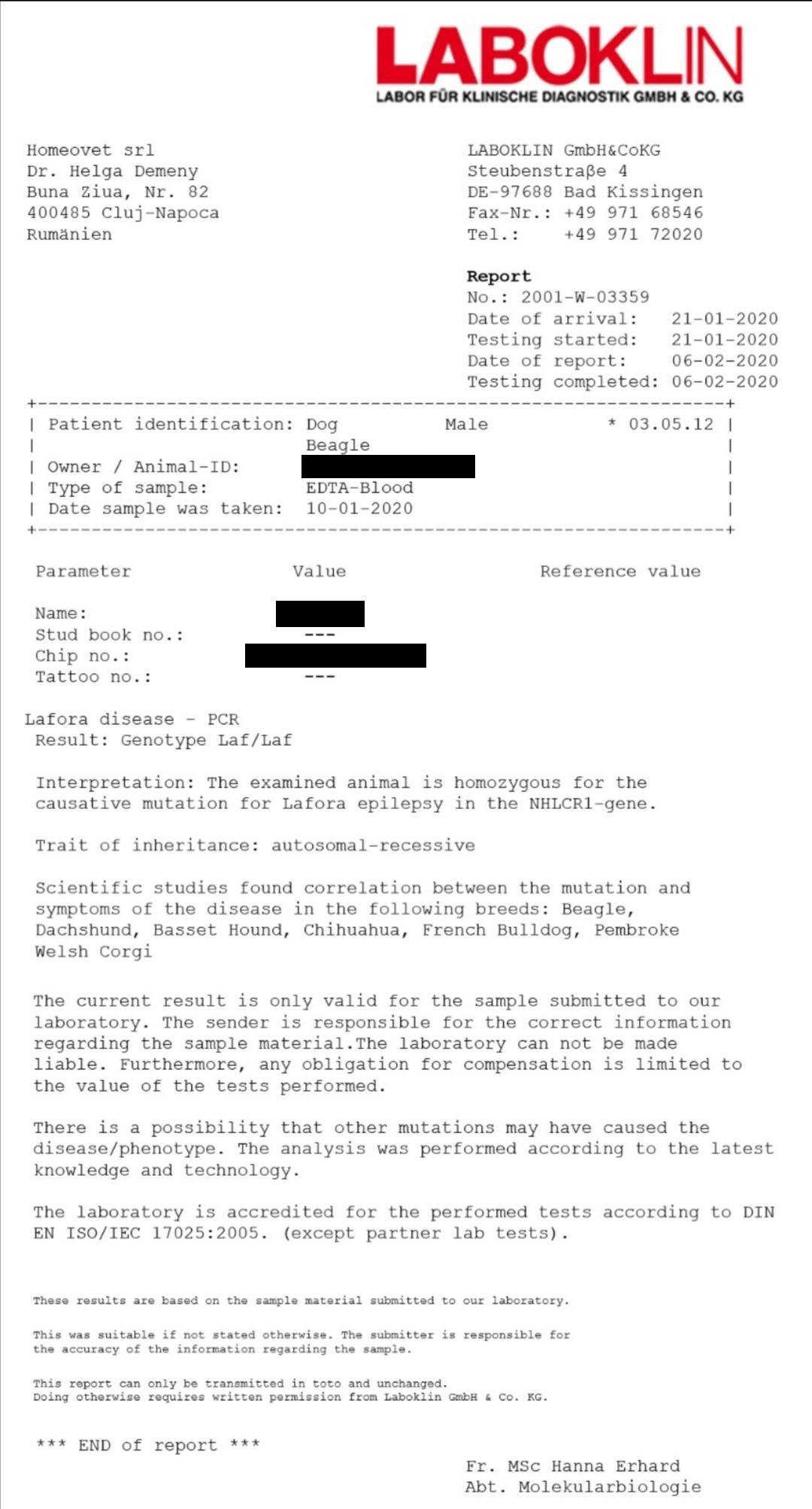

Supplement: Supplementary file 5 [file Image_2.JPEG]
